# Neo-Fs Index: A Novel Immunohistochemical Biomarker Panel Predicts Survival and Response to Anti-Angiogenetic Agents in Clear Cell Renal Cell Carcinoma

**DOI:** 10.3390/cancers13061199

**Published:** 2021-03-10

**Authors:** Jisup Kim, Jee-Young Park, Su-Jin Shin, Beom Jin Lim, Heounjeong Go

**Affiliations:** 1 Department of Pathology, Asan Medical Center, University of Ulsan College of Medicine, Seoul 05505, Korea; jspath@amc.seoul.kr; 2 Department of Pathology, Yonsei University College of Medicine, Seoul 03722, Korea; charm@yuhs.ac (S.-J.S.); bjlim@yuhs.ac (B.J.L.); 3 Department of Pathology, Kyungpook National University Medical Center, Kyungpook National University School of Medicine, Daegu 41944, Korea; pathpjy@naver.com

**Keywords:** clear cell renal cell carcinoma, frameshift insertion and deletion, immunohistochemical biomarker, anti-angiogenic agent response, prognosis

## Abstract

**Simple Summary:**

Although anti-angiogenetic agents (AAA) are mainstay treatments for clear cell renal cell carcinoma (ccRCC), there are very few histology-based predictive biomarkers applicable in routine clinical practice. Considering that frameshifts contribute to antitumor immunity and ccRCC harbors the highest indel proportion across tumors, we hypothesized that protein markers frequently mutated via frameshift indels could predict prognosis and response to AAA. We evaluated the prognostic impact of the individual protein markers and found five proteins showing independent prognostic value. Utilizing the five proteins, we developed an integrated biomarker—Neo-fs index. High Neo-fs index predicted better prognosis and AAA response. High Neo-fs index, which harbored greater single nucleotide variant and indel mutation, was also associated with antitumor immune gene signature. Neo-fs index could be a practical biomarker to improve risk stratification and predict AAA response in ccRCC patients.

**Abstract:**

*Background*: Frameshift indels have emerged as a predictor of immunotherapy response but were not evaluated yet to predict anti-angiogenetic agent (AAA) response or prognosis in clear cell renal cell carcinoma (ccRCC). *Methods*: Here, to develop biomarkers that predict survival and response to AAA, we evaluated the immunohistochemical expression of proteins whose genes frequently harbor frameshift indels in 638 ccRCC patients and correlated the individual and integrated markers with prognosis and AAA response. The mutational landscape was evaluated using targeted next-generation sequencing in 12 patients concerning protein markers. Immune gene signatures were retrieved from TCGA RNA seq data. *Results*: Five proteins (APC, NOTCH1, ARID1A, EYS, and filamin A) were independent adverse prognosticators and were incorporated into the Neo-fs index. Better overall, disease-specific and recurrence-free survival were observed with high Neo-fs index in univariate and multivariate survival analyses. Better AAA responses were observed with a high Neo-fs index, which reflected increased MHC class I, CD8+ T cell, cytolytic activity, and plasmacytoid dendritic cell signatures and decreased type II-IFN response signatures, as well as greater single-nucleotide variant (SNV) and indel counts. *Conclusions*: Neo-fs index, reflecting antitumor immune signature and more SNVs. and indels, is a powerful predictor of survival and AAA response in ccRCC.

## 1. Introduction

Frameshift insertions and deletions (indels) have gained considerable attention owing to the fact that numerous neoantigens with high specificity for the major histocompatibility complex (MHC) are produced upon the accumulation of such mutations, thereby contributing to antitumor immunity [1]. In particular, clear cell renal cell carcinoma (RCC), accounting for 65‒70% of all renal malignancies [2], harbors the highest proportion of coding indels across all tumor types; this phenomenon accounts for its immunogenicity and a high response to immunotherapy despite low non-synonymous single-nucleotide variant (SNV) burden [1]. Although frameshift indels first gained attention as a predictor of immune checkpoint inhibitor (ICI) response, a high load of frameshift indels also correlated with improved survival in ICI-naïve NSCLC patients [1], thereby suggesting extended applicability to the prediction of the response to therapeutic agents beyond ICIs as well as the prognosis.

Anti-angiogenetic agents (AAAs), including multi-targeted tyrosine kinase inhibitors (e.g., sunitinib, pazopanib, sorafenib, and axitinib) and VEGF-A monoclonal antibody (e.g., bevacizumab) [3], constitute a mainstay of treatment for advanced RCC patients [4]. With the recent incorporation of ICIs into a first-line systematic treatment of advanced RCC [5,6,7], biomarkers to predict AAA response have become more crucial for optimizing the treatment choice.

Turajlic et al. [1] have identified particular genes that are frequently mutated via frameshift indels and produce multiple high-affinity neoantigens in a pan-cancer analysis. We have hypothesized that analyzing the expression of these gene products, which might be reflective of frameshift indel burden, would affect prognosis and treatment response in clear cell RCC. Therefore, we investigated the proteins coded by genes that frequently harbor frameshift indels, resulting in the production of numerous neoantigens, and correlated them with prognosis as well as therapeutic responses to AAAs and immunotherapy in clear cell RCC patients in an attempt to develop novel prognostic and predictive biomarkers.

## 2. Results

### 2.1. Baseline Characteristics of the Study Population

The clinicopathological characteristics of the patients with clear cell RCC are depicted in Table 1. The mean age was 55.7 ± 12.0 years (median 56; range, 23–86) with a male to female ratio of 3.0. Partial nephrectomy was conducted in 340 (53.3%) patients and radical nephrectomy in 298 (46.7%) cases. The mean tumor size was 4.3 ± 2.8 cm (median 3.5; range, 0.3–21.0). Upon using the American Joint Committee on Cancer (AJCC) cancer staging system, the clear cell RCC population was found to consist of 473 patients with pT1 (74.1%), 23 patients with pT2 (3.6%), 136 patients with pT3 (21.3%), and six patients with pT4 disease (0.9%). Lymph node metastasis (pN1) was identified in 15 (2.4%) patients, and synchronous distant metastasis was present in 38 (6.0%) patients. The median follow-up duration was 70 months (range, 1‒98). With respect to adjuvant therapy, 65 (10.2%) patients received AAAs (sunitinib for 30 patients, pazopanib for 31 patients, and sorafenib for four patients as first-line), 38 (6.0%) patients received mammalian target of rapamycin (mTOR) inhibitors (everolimus for 35 patients and temsirolimus for three patients).

### 2.2. Immunohistochemical Marker Expression

The results of immunohistochemical marker expression, including the number and percentage of patients for each marker, are shown in Table 1.

### 2.3. Prognostic Impact of Immunohistochemical Markers

Univariate Cox regression analysis revealed that high expression of five markers—of the ten markers evaluated—including APC, NOTCH1, ARID1A, EYS, and filamin A, was associated with adverse prognosis (Table 2). Further, expression of four other markers, including FAT1, VHL, PTEN, and p53, were found to be associated with favorable prognosis in univariate Cox regression (Table 2); KMT2D did not show any prognostic significance (Table 2). In the AAA-recipient subgroup, expression of ARID1A was associated with shorter overall survival (OS) (hazard ratio (HR) 10.061; *p* = 0.033) and disease-specific survival (DSS) (HR 10.061; *p* = 0.033) (Table S1). In the AAA-recipient subgroup, EYS expression was also associated with shorter OS (HR 2.718; *p* = 0.002), DSS (HR 2.718; *p* = 0.002), and recurrence-free survival (RFS) (HR 2.848; *p* = 0.016) (Table S1).

Clinicopathological variables that were found to be significantly related to survival in univariate Cox regression were adjusted for in the multivariate Cox regression. Five markers (APC, NOTCH1, ARID1A, EYS, and filamin A) were found to be independent and significant predictors of poor survival when highly expressed (Table 3). High expression of APC (HR 2.717; *p* = 0.006) and Filamin A (HR 2.108; *p* = 0.048) was an independent prognostic factor for DSS. High NOTCH1 expression demonstrated independent prognostic significance for OS (HR 1.694; *p* = 0.028) and RFS (HR 2.021; *p* = 0.020). Tumors with high ARID1A expression showed adverse OS (HR 4.558; *p* = 0.005) and DSS (HR 6.303; *p* = 0.005). High EYS expression was also an independent prognostic factor for adverse OS (HR 1.806; *p*= 0.018) and DSS (HR 2.212; *p* = 0.012). Meanwhile, PTEN and p53 expression did not reveal independent prognostic significance (Table 3). In an AAA-recipient subgroup, ARID1A and EYS retained independent prognostic significance for adverse OS (ARID1A, HR 9.835, *p* = 0.041; EYS, HR 2.433; *p* = 0.009) and DSS (ARID1A, HR 9.835, *p* = 0.041; EYS, HR 2.433; *p* = 0.009) (Table S2).

### 2.4. Prognostic Impact of Neo-Fs Index and its Association with Clinicopathological Characteristics

In the survival analysis, we identified five independent prognostic markers—out of ten immunohistochemical markers—whose genes are susceptible to the incorporation of frameshift indels, thereby resulting in the production of numerous immunogenic neoantigens [1]. Considering that loss-of-function frameshift indels result in loss of corresponding protein expression, we hypothesized that an increased number of protein markers with low expression might exert a cumulative effect on survival. We created the Neo-fs index—defined as the number of marker proteins with low expression among the five independent prognosticators (APC, NOTCH1, ARID1A, EYS, and filamin A)—to develop a simple, practical biomarker with potential prognostic or predictive value reflective of tumor biology.

**Figure 1:**
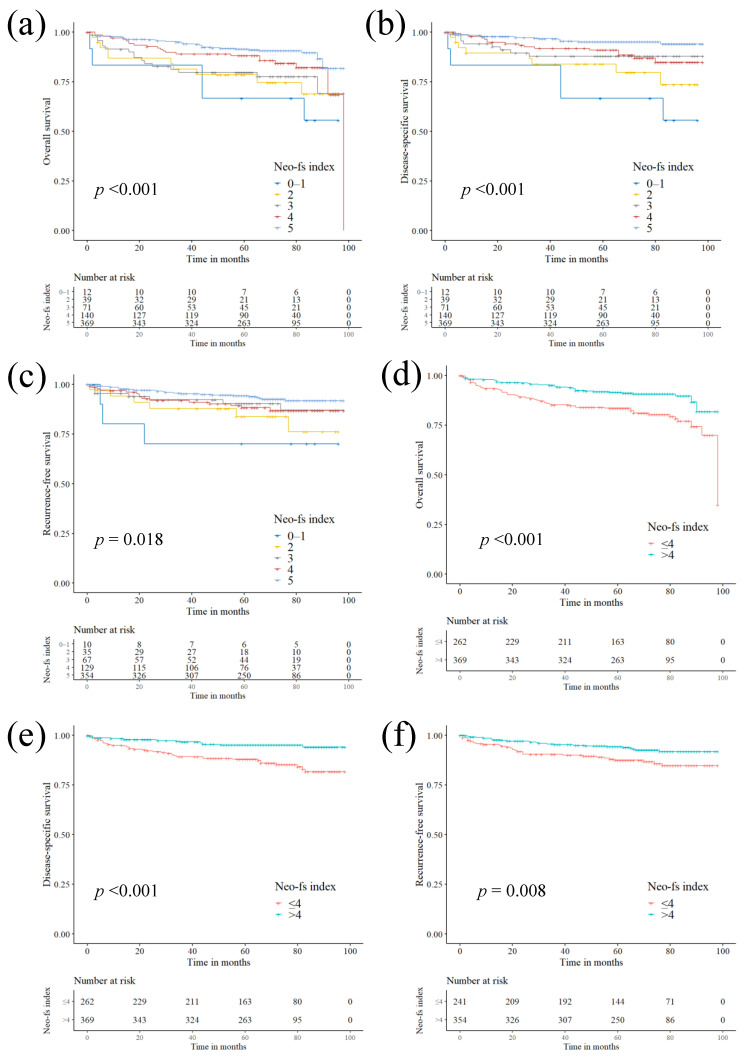
Kaplan‒Meier survival curves for patients with clear cell renal cell carcinoma based on the immunohistochemistry-based Neo-fs index. Neo-fs index—the number of highly expressed protein markers among APC, NOTCH1, ARID1A, EYS, and filamin A—demonstrated a cumulative favorable effect on overall survival (OS) (*p* < 0.001) (**a**), disease-specific survival (DSS) (*p* < 0.001) (**b**), and recurrence-free survival (RFS) (*p* = 0.018) (**c**) in patients with clear cell renal cell carcinoma. When stratified into Neo-fs index >4 and ≤4, Neo-fs index >4 was associated with favorable OS (*p* < 0.001) (**d**), DSS (*p* < 0.001) (**e**), and RFS (*p* = 0.008) (**f**).

Univariate Cox regression analysis revealed a gradual improvement in OS (*p*-for-trend < 0.001), DSS (*p*-for-trend < 0.001), and RFS (*p*-for-trend = 0.002) with higher Neo-fs index values (increased in a stepwise manner) (Table 2). Multivariate Cox regression revealed that the cumulative effect of high Neo-fs index values on favorable OS (*p*-for trend = 0.003) and DSS (*p*-for trend = 0.001) was retained (Table 3). The Kaplan‒Meier curve also revealed a gradual increase in OS (log-rank *p* < 0.001), DSS (log-rank *p* < 0.001), and RFS (log-rank *p* = 0.018) with higher values of the Neo-fs index (Figure 1). In the AAA-recipient subgroup, the cumulative effect higher Neo-fs index values on favorable survival was retained in univariate (OS, *p*-for trend = 0.004; DSS, *p*-for trend = 0.004; RFS, *p*-for trend = 0.022) and multivariate (OS, *p*-for trend = 0.001; DSS, *p*-for trend = 0.001) Cox regression analyses (Tables S1 and S2).

We next stratified the patients based on the Neo-fs index (low ≤4 vs. high >4) and revealed that high Neo-fs index was associated with longer OS (HR 0.461; *p* < 0.001; log-rank *p* < 0.001], DSS (HR 0.331; *p* < 0.001; log-rank *p* < 0.001] and RFS (HR 0.495; *p* = 0.010; log-rank *p* = 0.008] (Table 2, Figure 1). The prognostic significance of high Neo-fs index for better OS (HR 0.349; *p* = 0.002], DSS (HR 0.349; *p* = 0.002], and RFS (HR 0.347; *p* = 0.009] was retained in the AAA-recipient subgroup (Table S1). 

In multivariate analysis, high Neo-fs index (>4) was an independent prognostic factor for favorable OS (HR 0.595; *p*= 0.030), DSS (HR 0.430; *p*= 0.011), and RFS (HR 0.481; *p* = 0.020) (Table 3). The independent prognostic significance was retained for OS (HR 0.314; *p* = 0.003) and DSS (HR 0.372; *p* = 0.003) in the AAA-recipient subgroup (Table S2). 

We analyzed the clinicopathological characteristics of clear cell RCC patients based on the Neo-fs index (Table S3). Higher Neo-fs index was associated with International Society of Urological Pathology (ISUP) grade 1–2 (*p* < 0.001), pN0/pNx (*p* < 0.001), clear resection margin (*p* = 0.033), no AAA treatment (*p* = 0.009), and no mTOR inhibitor treatment (*p* = 0.016), although there was no difference with respect to sex, age, procedure, tumor size, pT stage, lymphovascular invasion, necrosis, or sarcomatoid change based on the Neo-fs index (Table S3).

### 2.5. Impact of Immunohistochemical Markers and Neo-fs Index on the Treatment Response

We analyzed the response to AAA and mTOR inhibitor, or lack thereof, with respect to the five individual independent prognosticators as well as the Neo-fs index (Table 4). Low expression of APC (overall response rate (ORR) 32.1% vs. 0%; *p* = 0.092), EYS (ORR 34.1% vs. 11.8%; *p* = 0.114), and Filamin A (ORR 33.3% vs. 7.7%; *p* = 0.088) exhibited a tendency for a better response to AAA. We also observed a cumulative effect of the five markers on the response to AAA; higher Neo-fs index values (increased in a stepwise manner) conferred an improved AAA response (*p* = 0.027). We discovered that patients with a high Neo-fs index (>4) (ORR 44.4%) exhibited significantly higher ORR to AAA than those with a low Neo-fs index (≤4) (ORR 14.7%) (*p* = 0.010). With respect to the mTOR inhibitor, low EYS expression exhibited a tendency for a better response to mTOR inhibitor (disease control rate (DCR) 38.1% vs. 0%; *p* = 0.075), while other markers failed to predict the response to mTOR inhibitor.

### 2.6. The Cancer Genome Atlas (TCGA) Gene Expression

**Figure 2:**
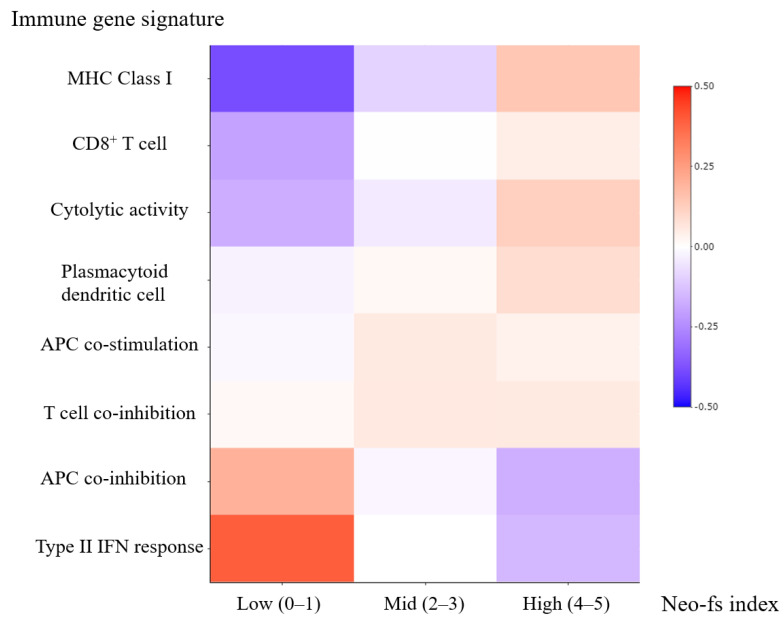
Heatmap depicting immune gene signatures according to RNA seq-based Neo-fs index in clear cell renal cell carcinoma. Immune gene signatures (mean z-scores) according to low- (0–1), mid- (2–3), and high (4–5) Neo-fs index were illustrated as a heatmap. Clear cell renal cell carcinoma samples with high Neo-fs index were enriched for genes associated with MHC Class I, CD8+ T cells, cytolytic activity, and plasmacytoid dendritic cell immune signatures. They were slightly enriched for immune gene signatures for APC co-stimulation and T cell co-inhibition. In contrast, they harbored decreased gene expressions associated with APC co-inhibition and type II-IFN response.

To evaluate the association between immune gene signature [8] and the genes of corresponding proteins, which showed independent prognostic significance, we evaluated the mRNA expression of 510 clear cell RCC patients using the available RNA seq data in the TCGA PanCancer Atlas dataset. Neo-fs index, initially developed using protein markers, was used to identify correlations with the RNAseq data. The Neo-fs index was evaluated as the number of genes with low expression among the five genes (APC, NOTCH1, ARID1A, EYS, and FLNA). The mean z-scores of each immune gene signature with respect to the Neo-fs index are illustrated in Figure 2. The mean value was compared between patients with a low Neo-fs index (0–1) and a high Neo-fs index (4–5) (Table S4). High Neo-fs index was associated with overexpression of immune gene signatures related to MHC class I (*p* < 0.001), CD8+ T cell (*p* = 0.008), cytolytic activity (*p* = 0.002), and plasmacytoid dendritic cells (*p* = 0.041), as compared with low Neo-fs index (Figure 2, Table S4). Immune gene signatures related to APC co-stimulation and T cell co-inhibition and were slightly enriched in high Neo-fs index but were not significant (*p* = 0.305 and 0.705 respectively) (Figure 2, Table S4). In contrast, a high Neo-fs index was associated with decreased expression of immune gene signatures associated with APC co-inhibition (*p* = 0.011) and type II-IFN response (*p* < 0.001) (Figure 2, Table S4).

### 2.7. Molecular Phenotype of Clear Cell Renal Cell Carcinoma and Neo-fs Index

**Figure 3:**
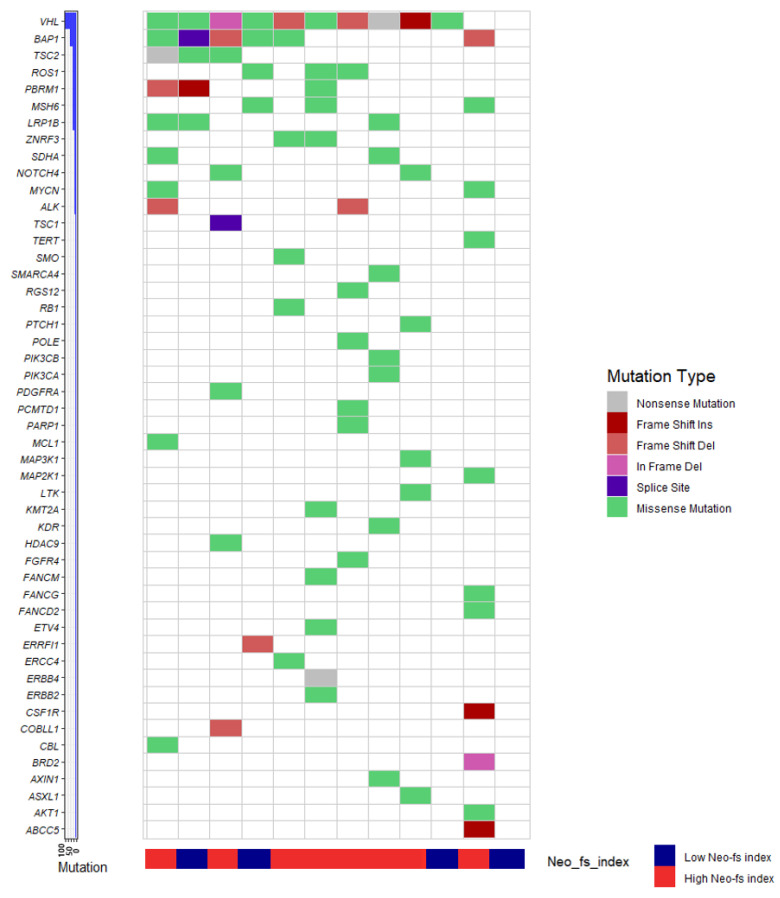
The landscape of somatic mutations in clear cell renal cell carcinoma, detected by targeted next-generation sequencing (NGS), is illustrated with Neo-fs index. Mutation types are annotated for each case by color bars according to the color panel on the right side of the image. The frequency of mutations for each gene is plotted in the left panel.

We have identified a total of 49 genes harboring somatic mutations in 12 cases of clear cell RCC by targeted next-generation sequencing (NGS) (Figure 3). The most frequently mutated gene was *VHL* (10 cases, 83.3%), followed by *BAP1* (six cases, 50.0%). The genes harboring frameshift insertion were *VHL*, *PBRM1*, *CSF1R*, and *ABCC5*, and the genes harboring frameshift deletion were *VHL, BAP1*, *TSC2*, *PBRM1*, *ALK*, *ERRFI1*, and *COBLL1*. The mean total mutation count was 6.67 ± 3.53. SNV counts (5.17 ± 2.89) were about four times higher than the total indel count (1.33 ± 1.56) (Table S5). The mean frameshift indel count was 1.08 ± 1.08 (Table S5). Eight cases with high Neo-fs index ( > 4) showed a higher count of total mutation (8.63 ± 1.85) and SNV (6.63 ± 2.00) compared with those in four cases with low Neo-fs index (0–2) (total mutation, 2.75 ± 2.63; SNV, 2.25 ± 2.06) (*p* = 0.001 and *p* = 0.005, respectively) (Table S5). Cases with high Neo-fs index (>4) also demonstrated a higher total indel count (1.88 ± 1.64) than those with low Neo-fs index (0–2) (0.25 ± 0.50) (*p* = 0.030), although only a weak tendency was observed regarding frameshift indel count (high Neo-fs index, 1.38 ± 1.19; low Neo-fs index, 0.50 ± 0.58) without statistical significance (*p* = 0.201) (Table S5).

## 3. Discussion

In this study, we investigated the prognostic value of individual protein markers—whose genes produce numerous frameshift neoantigens—and revealed that five protein markers (APC, NOTCH1, ARID1A, EYS, and filamin A) were independently associated with adverse prognosis when highly expressed in clear cell RCC tissues. Although EYS has not previously been investigated in the context of prognosis, the adverse prognostic impact of APC, NOTCH1, ARID1A, and filamin A has been reported in other cancer types. The expression of APC is associated with poor survival in pancreatic ductal adenocarcinoma [9]. NOTCH1 expression is associated with poor survival as well as advanced grade or TNM stage in hepatocellular carcinoma (HCC) and colorectal cancer [10]. In clear cell RCC, NOTCH1 expression is associated with advanced TNM stage and higher Fuhrman grade [11]. ARID1A loss—correlated with microsatellite instability and increased mutation burden across cancer types [12]—has shown favorable prognostic impact—is known to be associated with peritumoral lymphoid aggregates/tumor-infiltrating lymphocytes—in esophageal adenocarcinoma, suggesting the impact of neoantigen-induced immunogenicity on survival [13]. Filamin A expression has been reported to predict early recurrence as well as advanced T stage and grade in HCC [14]. Filamin A expression is also associated with shorter survival and early recurrence—as well as advanced TNM stage—in NSCLC [15].

With the incorporation of the independent prognosticators, we have developed a prognostic biomarker, named ‘Neo-fs index’, and revealed that a high Neo-fs index is independently associated with a decreased risk of adverse events. We are the first to demonstrate the prognostic value of the proteins—coded by genes producing numerous frameshift neoantigens [1]—as individual markers, as well as an integrated index. Previously, a high frameshift indel load and high clonal neoantigen burden [16] have been shown to be correlated with improved survival in NSCLC [1], although not for general prognostic impact in RCC.

We have also demonstrated a predictive value of Neo-fs index for the response to AAA in clear cell RCC in addition to the prognostic value. There was a gradual improvement in the AAA response according to the increasing Neo-fs index. Improved response to AAA was also achieved with low expression of APC, EYS, and filamin A as individual markers. A recent study has evaluated the clinical outcome of frameshift indel count in RCC, but the prognostic significance of frameshift indel count has not been demonstrated in AAA- recipients [17,18]. In contrast, we have demonstrated that a higher Neo-fs index, a marker which is based on the recurrent genes producing frameshift indel neoantigens, was associated with favorable OS and DSS in AAA-recipients, in addition to its predictive value for better AAA response.

To investigate the reason for favorable prognosis and better AAA response in cases with a higher Neo-fs index, we have analyzed the association between Neo-fs index and immune gene signature. Previously, a bi-directional link between angiogenesis and the immune system has been demonstrated. AAAs enhance the infiltration and function of effector T cells and diminish the infiltration and activity of immunosuppressive cells such as regulatory T cells and myeloid-derived suppressor cells (MDSCs) [19,20]. Also, MDSCs, activated by type II-IFN response, promote neo-angiogenesis and secrete angiogenetic factors, resulting in resistance to AAAs [21,22,23,24]. High Neo-fs index samples were enriched for genes linked to immune activation-associated signatures (e.g., plasmacytoid dendritic cells, cytolytic activity, MHC class I, and CD8+ T cell) had a reduced the gene signatures related to immune suppression such as type II-IFN response, which might have affected the response to AAA as well as favorable survival in general.

Moreover, we have evaluated the mutational landscape according to the Neo-fs index utilizing targeted NGS. Patients with a high Neo-fs index harbored higher counts of total mutation, SNV, and total indel than those with a low Neo-fs index. Although frameshift indel count was not significantly different between the two groups, the favorable prognosis, as well as enhanced T cell infiltration and function in the patients with high Neo-fs index, could be explained by higher SNV and total indel count because non-synonymous SNVs. are able to generate neoantigens that facilitate the generation of the T cell response [25,26,27]. In addition, a recent study in RCC patients has shown that SNVs. and in-frame indels generate neopeptides, and SNVs. produce as many immunogenic neoepitopes as frameshift indels, although neopeptides arising from frameshift indels showed less similarity to wild-type than those from SNVs. [28].

Interestingly, SNV was the most frequent mutation type in this study, although clear cell RCC is known to harbor the highest proportion of coding indels with low non-synonymous SNV burden [1]. However, Hansen et al. [28] has also shown that SNVs. accounted for the largest proportion of mutations leading to a greater number of predicted neopeptides.

Although genetic biomarkers (e.g., loss-of-function mutation of VHL) and molecular sub-classifications (e.g., ccrcc2/ccrcc3 from unsupervised transcriptome analysis) have shown their value in predicting better response to AAAs, none of the genetic or molecular biomarkers are in clinical practice [29]. On the other hand, histology-based biomarkers are more applicable for routine clinical use in terms of low time-consumption, cost-effectiveness, and ease of standardization regarding methodology and interpretation; nevertheless, there are scant histology-based biomarkers to predict response to AAAs in RCC [29]. Here we have revealed the predictive value of a novel histology-based marker, Neo-fs index, in clear cell RCC patients, which might address longstanding unmet needs for practical biomarkers reflecting tumor biology to guide the selection of patients sensitive to AAAs.

A limitation to this study is its single institutional design with retrospective nature. However, we analyzed a large series of cases with an adequate median follow-up duration and developed a simple and easily applicable protein-based biomarker—Neo-fs index—in clear cell RCC as a significant prognostic marker as well as a predictive marker for the response to AAA. Another limitation to this study is that we did not found a significant difference in frameshift indel burden according to Neo-fs index, probably due to the limitation inherent to the targeted sequencing compared with whole-genome sequencing. However, we have observed a weak tendency for higher frameshift indel count as well as significantly higher total mutation count, SNV count, and total indel count.

## 4. Materials and Methods

### 4.1. Case Selection

We selected 745 patients with clear cell RCC who had been treated at the Asan Medical Center between July 2011 and December 2013 based on our pathology records. Patients without available tissue or available clinical data were excluded (*n* = 107). Finally, a total of 638 patients who underwent partial nephrectomy (*n* = 340) or radical nephrectomy (*n* = 298) were selected for subsequent analysis. Electronic medical records were retrieved to obtain clinical data, including age, sex, surgical procedure, adjuvant therapy regimen, treatment response, distant metastasis, and survival outcomes. For targeted NGS, we selected separate 25 cases with clear cell RCC, for which the targeted NGS was performed between 2019 and 2020, and immunohistochemistry was available in the same paraffin block as NGS. This study was approved by our Institutional Review Board (approval number, 2019-0753).

### 4.2. Pathological Evaluation

Data retrieved from pathological reports included primary diagnosis, tumor size, nuclear grade, resection margin status, lymphovascular invasion, necrosis, sarcomatoid change, and pathological TNM stage, including the extent of the primary tumor, nodal, and distant metastasis. The nuclear grade was evaluated in accordance with the grading system recommended by the WHO/ISUP [2]. The pathological TNM stage was evaluated in accordance with the guidelines provided in the AJCC cancer staging manual (8th edition) [30].

### 4.3. Immunohistochemistry

Immunohistochemistry was performed for APC, NOTCH1, ARID1A, FAT1, VHL, EYS, KMT2D, Filamin A, PTEN, and p53 on 4-μm‒thick serial sections of tissue microarray (TMA) paraffin blocks containing two 3-mm‒diameter cores per sample. Tissues were deparaffinized at 76 °C for 4 min and rehydrated in a graded ethanol series. After blocking the endogenous peroxidase by incubating the sections with the OV PEROX inhibitor at 37 °C for 4 min, the sections were incubated for 16 min at 37 °C with the following primary antibodies: anti-APC antibody (rabbit, 1:50; GTX16794, Clone CC1; Genetex, Irvine, CA, USA), anti-Notch1 antibody (mouse, 1:200; ab52627, Clone EP1238Y; Abcam, Cambridge, UK), anti-ARID1A antibody (rabbit, 1:100; clone HPA005456; Sigma-Aldrich, Darmstadt, Germany), anti-FAT1 antibody (rabbit, 1:100; NBP1-84565; Novus Biologicals, Littleton, CO, USA), anti-VHL antibody (mouse, 1:2000; ab140989, clone OTI1E1; Abcam), anti-EYS/RP25 antibody (rabbit, 1:100; NBP1-90038; Novus Biologicals), anti-KMT2D antibody (rabbit, 1:100; NBP1-89123; Novus Biologicals), anti-filamin A antibody (mouse, 1:400; ab76289, clone EP1238Y; Abcam), anti-PTEN antibody (rabbit, 1:100; 138G6; Cell Signaling, Danvers, MA, USA), and anti-p53 antibody (mouse, 1:1000; DO-7; Dako, Glostrup, Denmark). Then, heat-induced antigen retrieval was performed. A Benchmark XT automatic staining system (Ventana Medical Systems, Tucson, AZ, USA) was utilized for the staining procedure, and reactions were visualized using OptiView DAB IHC Detection Kit (Ventana Medical Systems, Tucson, AZ, USA) and light counterstaining with hematoxylin. Lastly, the sections were dehydrated in ethanol and cleared in xylene. KMT2C, MAP2K1, and VPS13C were excluded from the evaluation because the expression level of these proteins as—identified by immunohistochemistry on formalin-fixed tissues—was not suitable for evaluation. When tumor cells were lost during the serial sectioning of the TMA paraffin blocks for immunohistochemistry or when there were no remaining samples to test the expression of some markers, the markers were analyzed in the order of priority using the cells as available.

Immunostaining of all markers was scored as negative (0), weak (1+), moderate (2+), and strong (3+) by two independent pathologists (JK and HG) who were blinded to the clinicopathological information. The expression of APC, NOTCH1, FAT1, EYS, filamin A, and PTEN was evaluated in the cytoplasm and the expression of ARID1A, VHL, KMT2D, and p53 was evaluated in the nucleus. Then the expression of each marker was stratified into low (0 for VHL, PTEN, and p53; 0–1 for APC, NOTCH1, FAT1, EYS, and KMT2D; 0–2 for ARIDA1, and Filamin A) or high (1–3 for VHL, PTEN, and p53; 2–3 for APC, NOTCH1, FAT1, EYS, and KMT2D; 3 for ARID1A, and filamin A) expression, respectively. Representative images of each marker are illustrated in Figure S1.

### 4.4. Outcome Measures

The primary endpoints were OS, DSS, RFS, and ORR to AAA and mTOR inhibitor. OS was defined as the duration from surgery until death from any cause or last follow-up. DSS was defined as the duration from surgery until death due to clear cell RCC or until the date of the last follow-up or death from any other causes. RFS was defined as the duration from surgery until the date of the first locoregional or distant relapse or until the last follow-up date. Patients with synchronous distant metastasis (*n* = 38) were excluded from RFS analysis. The ORR was defined as the percentage of patients with complete response (CR) or partial response (PR) out of all evaluable patients according to RECIST 1.1 criteria [8]. When ORR could not be evaluated, DCR, defined as the percentage of patients with CR, PR, or stable disease (SD) out of all evaluable patients, was evaluated instead [31].

### 4.5. TCGA Gene Expression Data

To evaluate the association between gene expression and immune signature, we retrieved RNA Seq V2 RSEM data (mRNA expression z-scores relative to diploid samples) from the Kidney Renal Cell Carcinoma (TCGA, PanCancer Atlas) dataset, available at cBioPortal for Cancer Genomics (https://www.cbioportal.org/, accessed on 25 October 2020). The gene sets used to define immune gene signature were adopted from Rooney and colleagues [8]. The mRNA expression of each gene was stratified into low or high expression by the median value. 

### 4.6. Targeted Next-Generation Sequencing

After determining tumor purity by review of matched hematoxylin and eosin (H&E) slides, genomic DNA (gDNA) was extracted from 2–5 sections (6 μm thick) of paraffin blocks in the marked area. Following de-paraffinization by treatment with xylene and ethanol, gDNA was isolated with the utilization of NEXprep FFPE Tissue kit (#NexK-9000; Geneslabs, Seongnam, Korea), in compliance with the manufacturer’s protocol [31]. Quantification of DNA was performed with the utilization of Qubit™ dsDNA HS Assay kit (Thermo Fisher Scientific, Waltham, MA, USA).

Targeted NGS was performed using the MiSeq platform (Illumina, San Diego, CA, USA) with OncoPanel AMC version 4.3 (OP_AMCv4.3) designed in-house by ASAN Center for Cancer Genome Discovery (ASAN-CCGD) to target a total of 328 genes (808 kb), including complete exonic sequence of 225 genes, 105 hot spots, and partial intronic sequence of six genes (Table S6) [31]. A DNA library was prepared by fragmentation of gDNA (200 ng) to an average of 250 bp by S1 enzyme (S1 method) [32], followed by sequential reactions of end repair, A tailing, and ligation of 50 ng of purified DNA with a TruSeq adaptor, by the utilization of a SureSelectXT Reagent kit (Agilent Technologies, Santa Clara, CA, USA). Each library was addressed with sample-specific barcodes (6 bp) and quantified using the Qubit kit. Eight libraries were pooled to yield a total of 750 ng for hybrid capture using an Agilent SureSelectXT custom kit (OP_AMCv3 RNA bait; Agilent Technologies). The concentration of enriched target was measured by quantitative polymerase chain reaction (qPCR; Kapa Biosystems, Woburn, MA, USA), and the DNA libraries which passed quality checks were loaded onto the MiSeq for paired-end sequencing.

Sequencing reads were aligned to the human reference genome (National Center for Biotechnology Information build 37) with the Burrows-Wheeler Aligner (0.5.9) with the default options [33]. PCR duplicates were removed using MarkDuplicates in the Picard package (Broad Institute, Cambridge, MA; available at http://broadinstitute.github.io/picard, accessed on 25 October 2020). De-duplicated reads were re-aligned at known indel positions using GATK IndelRealigner tool [34], followed by re-calibration of base qualities using GATK TableRecalibration tool. Somatic variant calling for SNVs. and short indels were performed with unmatched normal using the Mutect (version 1.1.7) and the SomaticIndelocator tool in GATK (Broad Institute) [34,35,36]. Common germline variants from the somatic variant candidate were filtered out using the Single Nucleotide Polymorphism database (dbSNP, build 141; found in >1% of samples), Exome Aggregation Consortium release 0.31 (threshold frequency 0.001), Korean Reference Genome database (KRGDB), and an in-house panel of normal variants [37,38]. Final somatic variants were annotated using the Variant Effect Predictor version 79 [39], which were then converted to maf file format by vcf2maf (version 1.6.12; available at https://github.com/mskcc/vcf2maf, accessed on 25 October 2020). False-positive variants were manually curated with the utilization of Integrative Genomics Viewer (version 2.4) [40].

Of the 25 cases enrolled for targeted NGS, cases with the lowest Neo-fs index available (2; four cases) and the highest Neo-fs index (5; eight cases) were selected for further evaluation to compare the mutational spectrum according to the Neo-fs index. 

### 4.7. Statistical Analysis

For descriptive variables, mean and standard deviation or median with range were utilized for continuous variables, and number and relative frequency were utilized for categorical variables. The means were compared using an independent two-sample *t*-test, and the frequencies were compared using chi-square or Fisher’s exact test. A univariate Cox proportional hazards regression analysis was conducted to identify the prognostically significant clinicopathological factors and immunohistochemical markers. A multivariate Cox proportional hazards regression was conducted to identify independent predictors of outcomes. For survival analysis, Kaplan‒Meier curves were also generated and were compared using the Log-rank test. The results are presented as HRs with a 95% confidence interval. All statistical analyses were performed using SPSS version 21.0 (IBM Corp, Armonk, NY, USA). All *p*-values presented are two-tailed, and *p*-values < 0.05 were considered significant.

## 5. Conclusions

In summary, we have introduced a novel prognostic and predictive biomarker—Neo-fs index—in clear cell RCC reflecting immune gene signatures as well as SNV and indel counts. Specifically, patients with a high Neo-fs index exhibited better survival and favorable response to AAA. The practical utilization of the Neo-fs index may pave the way for better-personalized care in patients with clear cell RCC.

## Figures and Tables

**Table 1:** Clinicopathological characteristics of the study population and immunohistochemistry results.

Clinicopathological Characteristics	N (%)	Immunohistochemistry	N (%)
**Sex**		**APC (0–1 vs. 2–3)**	
Male	480 (75.2%)	Low expression	548 (86.7%)
Female	158 (24.8%)	High expression	84 (13.3%)
**Age (years)**		**NOTCH1 (0–1 vs. 2–3)**	
<55 years	316 (49.5%)	Low expression	436 (69.0%)
≥55 years	322 (50.5%)	High expression	196 (31.0%)
**Procedure**		**ARID1A (0–2 vs. 3)**	
Partial nephrectomy	340 (53.3%)	Low expression	627 (99.1%)
Radical nephrectomy	298 (46.7%)	High expression	6 (0.9%)
WHO/ISUP nuclear grade *		**FAT1 (0–1 vs. 2–3)**	
1‒2	331 (51.9%)	Low expression	474 (74.9%)
3‒4	307 (48.1%)	High expression	159 (25.1%)
**Tumor size (cm)**		**VHL (0 vs. 1–3)**	
<4 cm	388 (60.8%)	Low expression	177 (28.0%)
≥4 cm	250 (39.2%)	High expression	455 (72.0%)
**pT stage**		**EYS (0–1 vs. 2–3)**	
pT1‒2	496 (77.7%)	Low expression	527 (83.0%)
pT3‒4	142 (22.3%)	High expression	108 (17.0%)
**pN stage**		**KMT2D (0–1 vs. 2–3)**	
pN0/pNx	623 (97.6%)	Low expression	296 (46.8%)
pN1	15 (2.4%)	High expression	337 (53.2%)
**Lymphovascular invasion**		**Filamin A (0–2 vs. 3)**	
Absent	537 (84.2%)	Low expression	579 (91.5%)
Present	101 (15.8%)	High expression	54 (8.5%)
**Resection margin**		**PTEN (0 vs. 1–3)**	
Clear	624 (97.8%)	Low expression	112 (17.6%)
Involved	14 (2.2%)	High expression	526 (82.4%)
**Necrosis**		**p53 (0 vs. 1–3)**	
Absent	538 (84.3%)	Low expression	36 (5.6%)
Present	100 (15.7%)	High expression	602 (94.4%)
**Sarcomatoid change**			
Absent	603 (94.5%)		
Present	35 (5.5%)		
**Anti-angiogenetic agent**			
Not received	573 (89.8%)		
Received	65 (10.2%)		
**mTOR inhibitor**			
Not received	600 (94.0%)		
Received	38 (6.0%)		

* WHO/International Society of Urological Pathology (ISUP) Grade 1, 28 cases; grade 2, 303 cases; grade 3, 245 cases; grade 4, 62 cases. Of the 638 samples, six samples lacked sufficient tumor cells for testing the expression of APC, NOTCH1, and VHL; five lacked sufficient tumor cells for testing the expression of ARID1A, FAT1, KMT2D, and Filamin A; three lacked sufficient tumor cells for testing the expression of EYS.

**Table 2:** Univariate analysis to identify a correlation between survival and clinicopathological factors and immunohistochemical results in patients receiving anti-angiogenic therapies.

Variables	Overall Survival		Disease-Specific Survival		Recurrence-Free Survival	
	HR (95% CI)	*p*	HR (95% CI)	*p*	HR (95% CI)	*p*
**Clinicopathologic variables**						
Female (vs. Male)	0.729 (0.430–1.238)	0.242	0.726 (0.376–1.402)	0.341	0.800 (0.422–1.515)	0.493
Age ≥ 55 years	3.328 (2.057–5.387)	**<0.001**	2.702 (1.516–4.817)	**0.001**	2.316 (1.322–4.059)	**0.003**
Radical nephrectomy (vs. partial nephrectomy)	3.797 (2.345–6.146)	**<0.001**	16.769 (6.069–46.335)	**<0.001**	3.915 (2.167–7.073)	**<0.001**
ISUP grade 3–4	4.052 (2.464–6.663)	**<0.001**	12.064 (4.818–30.210)	**<0.001**	5.385 (2.785–10.414)	**<0.001**
Tumor size ≥ 4 cm	5.622 (3.474–9.097)	**<0.001**	19.062 (7.610–47.747)	**<0.001**	4.818 (2.724–8.520)	**<0.001**
pT3–4	6.281 (4.117–9.584)	**<0.001**	16.709 (8.807–31.699)	**<0.001**	8.920 (5.205–15.289)	**<0.001**
pN1 (vs. pN0/pNx)	15.837 (8.688–28.868)	**<0.001**	26.214 (13.893–49.463)	**<0.001**	69.925 (25.878–188.944)	**<0.001**
Lymphovascular invasion	7.281 (4.777–11.097)	**<0.001**	12.505 (7.250–21.569)	**<0.001**	6.041 (3.522–10.360)	**<0.001**
Margin involvement	5.792 (2.793–12.010)	**<0.001**	7.757 (3.511–17.136)	**<0.001**	9.450 (4.038–22.113)	**<0.001**
Necrosis	7.462 (4.926–11.304)	**<0.001**	23.111 (12.436–42.951)	**<0.001**	8.777 (5.172–14.893)	**<0.001**
Sarcomatoid change	7.289 (4.416–12.031)	**<0.001**	12.974 (7.516–22.396)	**<0.001**	9.280 (4.792–17.970)	**<0.001**
AAA recipient	11.146 (7.334–16.938)	**<0.001**	36.948 (20.155–67.735)	**<0.001**	56.860 (32.589–99.207)	**<0.001**
mTOR inhibitor recipient	13.798 (8.881–21.438)	**<0.001**	32.525 (19.109–55.362)	**<0.001**	46.282 (24.568–87.191)	**<0.001**
**Immunohistochemistry**						
High APC expression	1.663 (0.979–2.827)	0.060	2.129 (1.143–3.966)	**0.017**	1.537 (0.774–3.049)	0.219
High NOTCH1 expression	1.806 (1.182–2.758)	**0.006**	2.029 (1.195–3.447)	**0.009**	1.835 (1.077–3.128)	**0.026**
High ARID1A expression	4.634 (1.675–12.820)	**0.003**	6.290 (1.954–20.251)	**0.002**	2.307 (0.319–16.687)	0.408
High FAT1 expression	0.659 (0.383–1.134)	0.132	0.415 (0.188–0.916)	**0.029**	0.627 (0.315–1.245)	0.182
High VHL expression	0.573 (0.375–0.877)	**0.010**	0.482 (0.286–0.814)	**0.006**	0.527 (0.307–0.904)	**0.020**
High EYS expression	2.416 (1.540–3.789)	**<0.001**	3.294 (1.911–5.676)	**<0.001**	1.710 (0.919–3.180)	0.090
High KMT2D expression	0.859 (0.562–1.313)	0.483	0.967 (0.566–1.653)	0.904	0.705 (0.413–1.205)	0.201
High Filamin A expression	2.439 (1.417–4.198)	**0.001**	3.826 (2.080–7.040)	**<0.001**	3.217 (1.659–6.236)	**0.001**
High PTEN expression	0.438 (0.280–0.686)	**<0.001**	0.284 (0.167–0.482)	**<0.001**	0.637 (0.337–1.207)	0.167
High p53 expression	0.719 (0.329–1.570)	0.408	0.387 (0.176–0.854)	**0.019**	0.361 (0.164–0.798)	**0.012**
**Neo-fs index**						
0–1	4.497 (1.759–11.498)	**0.002**	8.655 (3.206–23.369)	**<0.001**	4.715 (1.418–15.679)	**0.011**
2	2.811 (1.388–5.694)	**0.004**	4.553 (1.978–10.478)	**<0.001**	2.797 (1.143–6.843)	**0.024**
3	2.424 (1.337–4.395)	**0.004**	2.496 (1.085–5.741)	**0.031**	1.647 (0.710–3.822)	0.246
4	1.673 (0.981–2.853)	0.059	2.392 (1.220–4.691)	**0.011**	1.804 (0.946–3.439)	0.073
5 (reference)	1	-	1	-	1	-
**p-for trend**	0.690 (0.584–0.815)	**<0.001**	0.608 (0.499–0.741)	**<0.001**	0.711 (0.573–0.883)	**0.002**
**Neo-fs index**						
Low (≤4)	1	-	1	-	1	-
High (>4)	0.461 (0.301–0.708)	**<0.001**	0.331 (0.188–0.581)	**<0.001**	0.495 (0.291–0.844)	**0.010**

**Neo-fs index**: The number of markers with low expression among the five independent prognosticators (APC, NOTCH1, ARID1A, EYS, and Filamin A); The number of patients with the Neo-fs index 0–1, 2, 3, 4, and 5 was 12, 39, 71, 140, and 369, respectively. AAA, anti-angiogenetic agent; mTOR inhibitor, mammalian target of rapamycin (mTOR) inhibitor; CI, confidence interval.

**Table 3:** Multivariate analysis to identify a correlation between survival and clinicopathological factors and immunohistochemical results in patients receiving anti-angiogenic therapies.

Variables	Overall Survival (OS)		Disease-Specific Survival (DSS)		Recurrence-Free Survival (RFS)	
	HR (95% CI)	*p*	HR (95% CI)	*p*	HR (95% CI)	*p*
**Clinicopathologic variables**						
Age ≥ 55 years	3.005 (1.833–4.925)	**<0.001**	2.501 (1.365–4.585)	**0.003**	1.671 (0.923–3.027)	0.090
Radical nephrectomy (vs. partial nephrectomy)	0.915 (0.484–1.729)	0.784	1.919 (0.593–6.207)	0.276	0.811 (0.394–1.671)	0.571
ISUP grade 3–4	1.271 (0.704–2.296)	0.426	1.799 (0.634–5.105)	0.269	2.396 (1.098–5.227)	**0.028**
Tumor size ≥ 4 cm	2.374 (1.220–4.620)	**0.011**	3.516 (1.160–10.653)	**0.026**	2.348 (1.056–5.220)	**0.036**
pT3–4	0.874 (0.442–1.729)	0.699	0.824 (0.321–2.116)	0.687	1.545 (0.707–3.379)	0.276
pN1 (vs. pN0/pNx)	1.270 (0.585–2.757)	0.546	1.112 (0.503–2.458)	0.792	3.916 (1.075–14.266)	**0.038**
Lymphovascular invasion	1.537 (0.657–3.593)	0.322	1.409 (0.561–3.537)	0.465	2.162 (1.101–4.245)	**0.025**
Margin involvement	2.552 (1.441–4.519)	**0.001**	2.527 (1.222–5.225)	**0.012**	3.193 (1.033–9.870)	**0.044**
Necrosis	1.633 (0.885–3.012)	0.116	2.186 (0.948–5.038)	0.066	1.386 (0.642–2.994)	0.406
Sarcomatoid change	1.311 (0.710–2.420)	0.387	1.396 (0.739–2.636)	0.304	0.912 (0.392–2.122)	0.830
AAA recipient	2.796 (1.342–5.825)	**0.006**	6.642 (2.642–16.699)	**<0.001**	29.152 (13.253–64.125)	**<0.001**
mTOR inhibitor recipient	1.429 (0.696–2.934)	0.330	1.219 (0.586–2.537)	0.596	1.176 (0.540–2.562)	0.683
**Immunohistochemistry**						
High APC expression	NA	NA	2.717 (1.333–5.539)	**0.006**	NA	NA
High NOTCH1 expression	1.694 (1.057–2.714)	**0.028**	1.782 (0.963–3.298)	0.066	2.021 (1.116–3.659)	**0.020**
High ARID1A expression	4.558 (1.568–13.252)	**0.005**	6.303 (1.726–23.016)	**0.005**	NA	NA
High FAT1 expression	1.231 (0.690–2.197)	0.483	1.287 (0.542–3.053)	0.567	NA	NA
High VHL expression	1.131 (0.712–1.797)	0.601	1.273 (0.712–2.276)	0.415	1.003 (0.558–1.801)	0.992
High EYS expression	1.806 (1.108–2.945)	**0.018**	2.212 (1.188–4.119)	**0.012**	NA	NA
High Filamin A expression	1.524 (0.795–2.920)	0.204	2.108 (1.007–4.415)	**0.048**	1.243 (0.497–3.112)	0.642
High PTEN expression	0.977 (0.602–1.585)	0.924	0.999 (0.562–1.775)	0.998	NA	NA
High p53 expression	NA	NA	0.745 (0.325–1.707)	0.486	0.930 (0.364–2.377)	0.880
**Neo-fs index**						
0–1	2.099 (0.775–5.688)	0.145	3.135 (1.029–9.556)	**0.044**	1.840 (0.454–7.457)	0.393
2	2.285 (1.011–5.162)	**0.047**	4.494 (1.578–12.800)	**0.005**	2.935 (1.038–8.296)	**0.042**
3	2.774 (1.486–5.177)	**0.001**	3.007 (1.238–7.300)	**0.015**	1.475 (0.564–3.86)	0.428
4	1.128 (0.628–2.025)	0.688	1.665 (0.749–3.699)	0.211	2.265 (1.105–4.642)	0.026
5 (reference)	1	-	1	-	1	-
*p-for trend*	0.757 (0.632–0.907)	**0.003**	0.690 (0.552–0.863)	**0.001**	0.787 (0.615–1.007)	0.057
**Neo-fs index**						
Low (≤4)	1	-	1	-	1	-
High (>4)	0.595 (0.372–0.951)	**0.030**	0.430 (0.225–0.825)	**0.011**	0.480 (0.259–0.890)	**0.020**

**Neo-fs index**: The number of markers with low expression among the five independent prognosticators (APC, NOTCH1, ARID1A, EYS, and Filamin A); The number of patients with the Neo-fs index 0–1, 2, 3, 4, and 5 was 12, 39, 71, 140, and 369, respectively. AAA, anti-angiogenetic agent; mTOR inhibitor, mammalian target of rapamycin (mTOR) inhibitor; CI, confidence interval.

**Table 4:** Association between immunohistochemical expression and the response to therapy.

Variables	Anti-Angiogenic Agent			mTOR Inhibitor		
	PR/SD,PD	ORR	*p*	SD/PD	DCR	*p*
Low APC	17/36	32.1%	0.092	7/18	28.0%	0.999
High APC	0/8	0%		1/2	33.3%	
Low NOTCH1	12/26	31.6%	0.406	5/14	26.3%	0.573
High NOTCH1	5/18	21.7%		3/6	33.3%	
Low ARID1A	17/44	27.9%	0.999	8/19	29.6%	0.999
High ARID1A	0/1	0%		0/1	0.0%	
Low EYS	15/29	34.1%	0.114	8/13	38.1%	0.075
High EYS	2/15	11.8%		0/7	0%	
Low Filamin A	16/32	33.3%	0.088	6/16	27.3%	0.999
High Filamin A	1/12	7.7%		2/4	33.3%	
**Indel signatures (positive number)**						
0–1	0/3	0%	**0.027**	0/2	0%	0.742
2	1/4	20.0%		0/1	0%	
3	1/8	11.1%		2/1	66.7%	
4	3/14	17.6%		2/6	25.0%	
5	12/15	44.4%		4/10	28.6%	
**Indel signatures (positive number)**						
Neo-fs index ≤ 4	5/29	14.7%	**0.010**	4/10	28.6%	0.999
Neo-fs index > 4	12/15	44.4%		4/10	28.6%	

ORR: Overall response rate (CR + PR/all evaluable patients); DCR: Disease control rate (CR + PR + SD/all evaluable patients); mTOR inhibitor, mammalian target of rapamycin (mTOR) inhibitor.

## Data Availability

The data presented in this study are available on request from the corresponding author. The data are not publicly available due to privacy or ethical restrictions.

## Supplementary Materials

The following are available online at https://www.mdpi.com/2072-6694/13/6/1199/s1, Figure S1: Representative images depicting high and low expression of APC, NOTCH1, ARID1A, FAT1, VHL, EYS, KMT2D, Filamin A, PTEN, and p53 in clear cell renal cell carcinoma; Table S1: Univariate analysis to identify a correlation between survival and clinicopathological factors and immunohistochemical results in patients receiving anti-angiogenic therapies; Table S2: Multivariate analysis to identify a correlation between survival and clinicopathological factors and immunohistochemical results in patients receiving anti-angiogenic therapies; Table S3: Clinicopathological characteristics of the study population based on the Neo-fs index; Table S4: Immune signature (mean z-scores) in clear cell renal cell carcinoma based on the Neo-fs index; Table S5: Comparison of mutational landscape in clear cell renal cell carcinoma based on Neo-fs index; Table S6. The list of genes targeting in the OncoPanel AMC version 4.3.
