# Supplementary material for: Neo-Fs Index: A Novel Immunohistochemical Biomarker Panel Predicts Survival and Response to Anti-Angiogenetic Agents in Clear Cell Renal Cell Carcinoma
Source: Cancers (Basel). 2021 Mar 10;13(6):1199. doi: 10.3390/cancers13061199 (PMC8000111; doi:10.3390/cancers13061199)
Supplement: Supplementary file 1 [file cancers-13-01199-s001.pdf]

cancers

Supplementary Materials

Neo-fs index: a novel immunohistochemical biomarker panel predicts survival and response to anti-angiogenetic agents in clear cell renal cell carcinoma

Jisup Kim, Jee-Young Park, Su-Jin Shin, Beom Jin Lim and Heounjeong Go

Table S1. Univariate analysis to identify a correlation between survival and clinicopathological factors and immunohistochemical results in patients receiving anti-angiogenic therapies

	Overall survival (OS)		Disease-specific survival (DSS)		Recurrence-free survival (RFS)	
	HR (95% CI)	*p*	HR (95% CI)	*p*	HR (95% CI)	*p*
**Clinicopathological variables**						
Female (vs Male)	0.874 (0.402-1.899)	0.734	0.874 (0.402-1.899)	0.734	1.049 (0.444-2.479)	0.914
Age ≥55 years	1.171 (0.624-2.199)	0.623	1.171 (0.624-2.199)	0.623	1.334 (0.596-2.985)	0.483
Radical nephrectomy (vs partial nephrectomy)	1.893 (0.675-5.304)	0.225	1.893 (0.675-5.304)	0.225	1.169 (0.502-2.722)	0.717
ISUP grade 3–4	3.452 (0.834-14.290)	0.087	3.452 (0.834-14.290)	0.087	2.294 (0.869-6.058)	0.094
Tumor size ≥4 cm	2.618 (1.026-6.679)	**0.044**	2.618 (1.026-6.679)	**0.044**	1.739 (0.807-3.749)	0.158
pT3–4	1.761 (0.807-3.844)	0.156	1.761 (0.807-3.844)	0.156	1.365 (0.649-2.871)	0.411
pN1 (vs pN0/pNx)	3.094 (1.566-6.113)	**0.001**	3.094 (1.566-6.113)	**0.001**	8.198 (2.323-28.932)	**0.001**
Lymphovascular invasion	2.541 (1.304-4.953)	**0.006**	2.541 (1.304-4.953)	**0.006**	2.307 (1.062-5.012)	**0.035**
Margin involvement	1.514 (0.634-3.615)	0.350	1.514 (0.634-3.615)	0.350	3.017 (1.146-7.944)	**0.025**
Necrosis	1.662 (0.796-3.472)	0.176	1.662 (0.796-3.472)	0.176	1.771 (0.865-3.626)	0.118
Sarcomatoid change	2.309 (1.247-4.278)	**0.008**	2.309 (1.247-4.278)	**0.008**	1.050 (0.449-2.455)	0.910
mTOR inhibitor recipient	2.781 (1.421-5.444)	**0.003**	2.781 (1.421-5.444)	**0.003**	1.869 (0.902-3.872)	0.092
**Immunohistochemistry**						
High APC expression	1.549 (0.685-3.505)	0.293	1.549 (0.685-3.505)	0.293	1.589 (0.600-4.210)	0.351
High NOTCH1 expression	1.814 (0.981-3.355)	0.058	1.814 (0.981-3.355)	0.058	1.951 (0.922-4.127)	0.080
High ARID1A expression	10.061 (1.211-83.584)	**0.033**	10.061 (1.211-83.584)	**0.033**	NA	NA
High FAT1 expression	0.814 (0.289-2.292)	0.697	0.814 (0.289-2.292)	0.697	0.918 (0.274-3.075)	0.890
High VHL expression	0.698 (0.382-1.273)	0.241	0.698 (0.382-1.273)	0.241	1.086 (0.519-2.273)	0.826
High EYS expression	2.718 (1.451-5.092)	**0.002**	2.718 (1.451-5.092)	**0.002**	2.848 (1.214-6.679)	**0.016**
High KMT2D expression	1.577 (0.846-2.940)	0.152	1.577 (0.846-2.940)	0.152	2.102 (0.939-4.707)	0.071
High Filamin A expression	1.928 (0.982-3.786)	0.057	1.928 (0.982-3.786)	0.057	2.213 (0.954-5.132)	0.064
High PTEN expression	0.717 (0.386-1.334)	0.294	0.717 (0.386-1.334)	0.294	1.412 (0.631-3.160)	0.402
High p53 expression	1.363 (0.487-3.818)	0.556	1.363 (0.487-3.818)	0.556	1.543 (0.531-4.480)	0.425

**Neo-fs index**	**Log-rank p**	**0.001**	**Log-rank p**	**0.001**	**Log-rank p**	0.096
0–1	1.953 (0.550-6.934)	0.300	1.953 (0.550-6.934)	0.300	3.188 (0.837-12.144)	0.089
2	6.678 (2.443-18.252)	**<0.001**	6.678 (2.443-18.252)	**<0.001**	3.101 (0.660-14.567)	0.152
3	3.370 (1.312-8.655)	**0.012**	3.370 (1.312-8.655)	**0.012**	2.739 (0.982-7.641)	0.054
4	2.373 (1.077-5.230)	**0.032**	2.373 (1.077-5.230)	**0.032**	2.862 (1.076-7.612)	**0.035**
5 (reference)	1	-	1	-	1	-
p-for trend	0.738 (0.599-0.909)	**0.004**	0.738 (0.599-0.909)	**0.004**	0.733 (0.562-0.957)	**0.022**
**Neo-fs index**						
Low (≤4)	1	-	1	-	1	-
High (>4)	0.349 (0.177-0.688)	**0.002**	0.349 (0.177-0.688)	**0.002**	0.347 (0.157-0.765)	**0.009**

Neo-fs index: The number of markers with low expression among the five independent prognosticators (APC, NOTCH1, ARID1A, EYS, and Filamin A)

The number of patients with the Neo-fs index 0–1, 2, 3, 4, and 5 was three, six, nine, 17, and 28, respectively

* mTOR inhibitor, mammalian target of rapamycin (mTOR) inhibitor; CI, confidence interval

Table S2. Multivariate analysis to identify a correlation between survival and clinicopathological factors and immunohistochemical results in patients receiving anti-angiogenic therapies

	Overall survival (OS)		Disease-specific survival (DSS)		Recurrence-free survival (RFS)	
	**HR (95% CI)**	***p***	**HR (95% CI)**	***p***	**HR (95% CI)**	***p***
**Clinicopathological variables**						
Tumor size ≥ 4 cm	1.429 (0.516-3.962)	0.492	1.429 (0.516-3.962)	0.492	NA	NA
pN1 (vs pN0/pNx)	1.702 (0.756-3.836)	0.199	1.702 (0.756-3.836)	0.199	4.681 (1.160-18.894)	**0.030**
Lymphovascular invasion	1.500 (0.698-3.225)	0.299	1.500 (0.698-3.225)	0.299	1.798 (0.750-4.312)	0.189
Margin involvement	NA	NA	NA	NA	2.450 (0.877-6.845)	0.087
Sarcomatoid change	1.496 (0.746-3.000)	0.257	1.496 (0.746-3.000)	0.257	NA	NA
mTOR inhibitor recipient	1.977 (0.947-4.124)	0.069	1.977 (0.947-4.124)	0.069	NA	NA
**Immunohistochemistry**						
High ARID1A expression	9.835 (1.100-87.939)	**0.041**	9.835 (1.100-87.939)	**0.041**	2.431 (0.964-6.130)	0.060
High EYS expression	2.433 (1.243-4.762)	**0.009**	2.433 (1.243-4.762)	**0.009**	NA	NA
**Neo-fs index**						
0–1	2.987 (0.766-11.646)	0.115	2.987 (0.766-11.646)	0.115	0.917 (0.131-6.431)	0.930
2	10.806 (2.897-40.308)	**<0.001**	10.806 (2.897-40.308)	**<0.001**	0.616 (0.140-2.697)	0.520
3	3.619 (1.356-9.659)	**0.010**	3.619 (1.356-9.659)	**0.010**	0.751 (0.170-3.305)	0.704
4	2.632 (0.995-6.962)	0.051	2.632 (0.995-6.962)	0.051	0.354 (0.086-1.460)	0.151
5 (reference)	1	-	1	-	1	-
p-for trend	0.662 (0.516-0.850)	**0.001**	0.662 (0.516-0.850)	**0.001**	0.778 (0.573-1.057)	0.108
**Neo-fs index**						
Low (≤4)	1	**-**	1	**-**	1	**-**
High (>4)	0.314 (0.146-0.679)	**0.003**	0.314 (0.146-0.679)	**0.003**	0.473 (0.198-1.132)	0.093

Neo-fs index: The number of markers with low expression among the five independent prognosticators (APC, NOTCH1, ARID1A, EYS, and Filamin A) # The number of patients with the Neo-fs index 0–1, 2, 3, 4, and 5 was three, six, nine, 17, and 28, respectively

* mTOR inhibitor, mammalian target of rapamycin (mTOR) inhibitor; CI, confidence interval.

Table S3. Clinicopathological characteristics of the study population based on the Neo-fs index

Clinicopathological variables	Neo-fs index					*p*
	0–1 (n=12)	2 (n=39)	3 (n=71)	4 (n=140)	5 (n=369)	
**Sex**						0.416
Male	9 (75.0%)	32 (82.1%)	57 (80.3%)	99 (70.7%)	277 (75.1%)	
Female	3 (25.0%)	7 (17.9%)	14 (19.7%)	41 (29.3%)	92 (24.9%)	
**Age (years)**						0.460
< 55 years	5 (41.7%)	21 (53.8%)	34 (47.9%)	62 (44.3%)	192 (52.0%)	
≥55 years	7 (58.3%)	18 (46.2%)	37 (52.1%)	78 (55.7%)	177 (48.0%)	
**Procedure**						0.425
Partial nephrectomy	6 (50.0%)	16 (41.0%)	41 (57.7%)	76 (54.3%)	200 (54.2%)	
Radical nephrectomy	6 (50.0%)	23 (59.0%)	30 (42.3%)	64 (45.7%)	169 (45.8%)	
**ISUP grade**						**<0.001**
1–2	3 (25.0.%)	10 (25.6%)	34 (47.9%)	61 (43.6%)	221 (59.9%)	
3–4	9 (75.0%)	29 (74.4%)	37 (52.1%)	79 (56.4%)	148 (40.1%)	
**Tumor size (cm)**						0.873
<4 cm	6 (50.0%)	23 (59.0%)	45 (63.4%)	87 (62.1%)	223 (60.4%)	
≥4 cm	6 (50.0%)	16 (41.0%)	26 (36.6%)	53 (37.9%)	146 (39.6%)	
**T stage**						0.394
pT1–2	7 (58.3%)	29 (74.4%)	58 (81.7%)	109 (77.9%)	290 (78.6%)	
pT3–4	5 (41.7%)	10 (25.6%)	13 (18.3%)	31 (22.1%)	79 (21.4%)	
**N stage**						**<0.001**
pN0/pNx	11 (91.7%)	35 (89.7%)	70 (98.6%)	133 (95.0%)	368 (99.7%)	
pN1	1 (8.3%)	4 (10.3%)	1 (1.4%)	7 (5.0%)	1 (0.3%)	
**Lymphovascular invasion**						0.074
Absent	8 (66.7%)	29 (74.4%)	64 (90.1%)	114 (81.4%)	318 (86.2%)	
Present	4 (33.3%)	10 (25.6%)	7 (9.9%)	26 (18.6%)	51 (13.8%)	
**Resection margin**						**0.033**
Clear	10 (83.3%)	38 (97.4%)	69 (97.2%)	137 (97.9%)	363 (98.4%)	
Involved	2 (16.7%)	1 (2.6%)	2 (2.8%)	3 (2.1%)	6 (1.6%)	
**Necrosis**						0.093
Absent	8 (66.7%)	32 (82.1%)	59 (83.1%)	116 (82.9%)	318 (86.2%)	
Present	4 (33.3%)	7 (17.9%)	12 (16.9%)	24 (17.1%)	51 (13.8%)	
**Sarcomatoid change**						0.133
Absent	10 (83.3%)	37 (94.9%)	67 (94.4%)	130 (92.9%)	354 (95.9%)	
Present	2 (16.7%)	2 (5.1%)	4 (5.6%)	10 (7.1%)	15 (4.1%)	
**Anti-angiogenetic agent**						**0.009**
Not received	9 (75.0%)	33 (84.6%)	62 (87.3%)	123 (87.9%)	341 (92.4%)	
Received	3 (25.0%)	6 (15.4%)	9 (12.7%)	17 (12.1%)	28 (7.6%)	
**mTOR inhibitor**						**0.016**
Not received	10 (83.3%)	35 (89.7%)	65 (91.5%)	131 (93.6%)	353 (95.7%)	
Received	2 (16.7%)	4 (10.3%)	6 (8.5%)	9 (6.4%)	16 (4.3%)	

Neo-fs index: The number of markers with low expression among the five independent prognosticators (APC, NOTCH1, ARID1A, EYS, and Filamin A)

# Of the 638 samples, seven lacked sufficient tumor cells for testing the expression of at least one marker among APC, NOTCH1, ARID1A, EYS, and Filamin A; these samples were excluded, and 631 samples were analyzed.

* mTOR inhibitor, mammalian target of rapamycin (mTOR) inhibitor

Table S4. Immune signature (mean z-scores) in clear cell renal cell carcinoma based on the Neo-fs index

**Immune gene signature**	**Neo-fs index**		**p**
	**Low (0–1) (mean±SD)**	**High (4–5) (mean±SD)**	
MHC Class I	-0.39±0.59	0.15±0.93	**<0.001**
CD8+ T cells	-0.20±0.65	0.05±0.99	**0.008**
Cytolytic activity	-0.18±0.63	0.12±1.06	**0.002**
pDCs	-0.03±0.42	0.09±0.59	**0.041**
Co-stimulation, APC	-0.02±0.37	0.03±0.46	0.305
Co-inhibition, T cell	0.02±1.13	0.06±0.69	0.705
Co-inhibition, APC	0.20±1.97	-0.17±0.52	**0.011**
Type II IFN Response	0.39±0.75	-0.16±0.55	**<0.001**

Neo-fs index: The number of markers with low expression among the five independent prognosticators (APC, NOTCH1, ARID1A, EYS, and Filamin A); **SD**: standard deviation; **pDCs**: plasmacytoid dendritic cells

Table S5. Comparison of mutational landscape in clear cell renal cell carcinoma based on Neo-fs index

	**Neo-fs index**			**p**
	**Total**	**Low (0–2)**	**High (>4)**	
Total mutation count	6.67±3.53	2.75±2.63	8.63±1.85	**0.001**
Single nucleotide variant count	5.17±2.89	2.25±2.06	6.63±2.00	**0.005**
Total indel count	1.33±1.56	0.25±0.50	1.88±1.64	**0.030**
Frameshift indel count	1.08±1.08	0.50±0.58	1.38±1.19	0.201

Neo-fs index: The number of markers with low expression among the five independent prognosticators (APC, NOTCH1, ARID1A, EYS, and Filamin A); **SD**: standard deviation

Table S6. The list of genes targeting in the OncoPanel AMC version 4.3

	**1) Entire exonic sequence were included for detection of Base Substitution, Insertions/Deletions, and Copy Number Alterations**
	ABL1, ABL2, AKT1, AKT2, AKT3, ALK, APC, AR, ARAF, ARID1A, ARID1B, ARID2, ASXL1, ATM, ATR, ATRX, AURKA, AURKB, AURKC, AXIN1, AXL, BAP1, BARD1, BRAF, BRCA1, BRCA2, BRD2, BRD3, BRD4, BRIP1, CBFB, CCND1, CCND2, CCND3, CCNE1, CD274, CDH1, CDK12, CDK4, CDK6, CDKN1A, CDKN1B, CDKN2A, DKN2B, CDKN2C, CEBPA, CHEK2, CREBBP, CSF1R, CTNNB1, DDR1, DDR2, DDX3X, DNMT3A, DOT1L, DPYD, EGFR, EPHA3, EPHB4, ERBB2, ERBB3, ERBB4, ERCC2, ERCC4, ERG, ERRFI1, ESR1, ETV1, ETV4, ETV5, ETV6, EWSR1, EZH2, FAM175A, FANCA, FANCB, FANCC, FANCD2, FANCE, FANCF, FANCG, FANCI, FANCL, FANCM, FBXW7, FGF19, FGF4, FGFR1, FGFR2, FGFR3, FGFR4, FLCN, FLT1, FLT3, FLT4, FOXL2, FUBP1, GATA2, GEN1, GNA11, GNAQ, GNAS, H3F3A, HDAC9, HGF, HLA-A, HLA-B, HLA-C, HLA-DRB1, HNF1A, HRAS, IDH1, IDH2, IGF1R, IGF2, JAK1, JAK2, JAK3, KDR, KIT, KMT2A, KRAS, LRP1B, LTK, MAP2K1, MAP2K2, MAP2K4, MAP3K1, MAP3K4, MAPK1, MAPK3, MAPK8, MCL1, MDM2, MDM4, MED12, MEN1, MET, MITF, MLH1, MPL, MRE11A, MSH2, MSH6, MTAP, MTOR, MYC, MYCN, NBN, NF1, NF2, NFKBIA, NKX2-1, NOTCH1, NOTCH2, NOTCH3, NOTCH4, NRAS, NTRK1, NTRK2, NTRK3, NUTM1, PALB2, PARP1, PBRM1, PDGFB, PDGFRA, DGFRB, PIK3CA, PIK3CB, PIK3CD, PIK3R1, PIK3R2, PMS2, POLE, PPARG, PTCH1, PTCH2, PTEN, PTPN11, RAD50, RAD51, RAD51C, RAD51D, RAF1, RARA, RB1, RET, RICTOR, RNF43, ROS1, RSPO1, RSPO2, RUNX1, SDHA, SDHB, SDHC, SDHD, SETD2, SLX4, SMAD2, SMAD4, SMARCA4, SMARCB1, SMO, SOX2, SOX9, SPOP, SRC, STK11, SYK, TERT, TET2, TMPRSS2, TOP1, TOP2A, TP53, TSC1, TSC2, UBE2T, VHL, WT1, XPO1, XRCC2, ZNRF3
Genes	**2) Partial intronic sequence were included for detection of Rearrangements**
	ALK, EGFR, NTRK1, RET, ROS1, BRAF
	**3) Hot spot was included for detection of Base Substitution, Insertions/Deletions**
	A1BG, ABCC5, ACVR2A, ADAMTS18, ADNP, AKAP7, AP1S1, ARV1, ASH1L, BAX, CASD1, CBX4, CCDC73, CD3G, CDH26, CEBPZ, CENPV, CKAP2, CLOCK, COBLL1, CPEB2, CRIPAK, DLC1, DNAH12, DOCK3, DPAGT1, DYNC1I2, EBPL, EPPK1, FBXL3, FGFBP1, FMN2, FRG2B, FXR1, GRIN3B, GTPBP2, HIAT1, IMPA1, INO80E, IRS1, KCTD16, KIAA1919, KRT32, LIPT1, MADCAM1, MVK, MYO1A, NIPA2, NNAT, NOS3, NOTCH2NL, NUDT7, OR4M2, PABPC1, PCBP1, PCDHB16, PCMTD1, PREX2, PRIM2, RASA4, RBBP8, RGS12, RHOA, RUFY2, SEC63, SF3B1, SLC23A2, SPRR3, SSTR4, STAMBPL1, STAU2, SULT6B1, SYNJ2, TAS2R19, TAS2R31, TCF7L2, TEAD2, TMEM60, TMPRSS13, TPSD1, WDR55, WDR87, ZFP37, ZNF141, ZNF563, BTK, CBL, IFITM1, IFITM3, KNSTRN, MAX, NFE2L2, PPP2R1A, PTPN11, RAC1, STAT3, U2AF1, KLF4, CIC, GATA3, KIF5B, ADCYAP1, IMPA2, MIR3648-1, DX3X

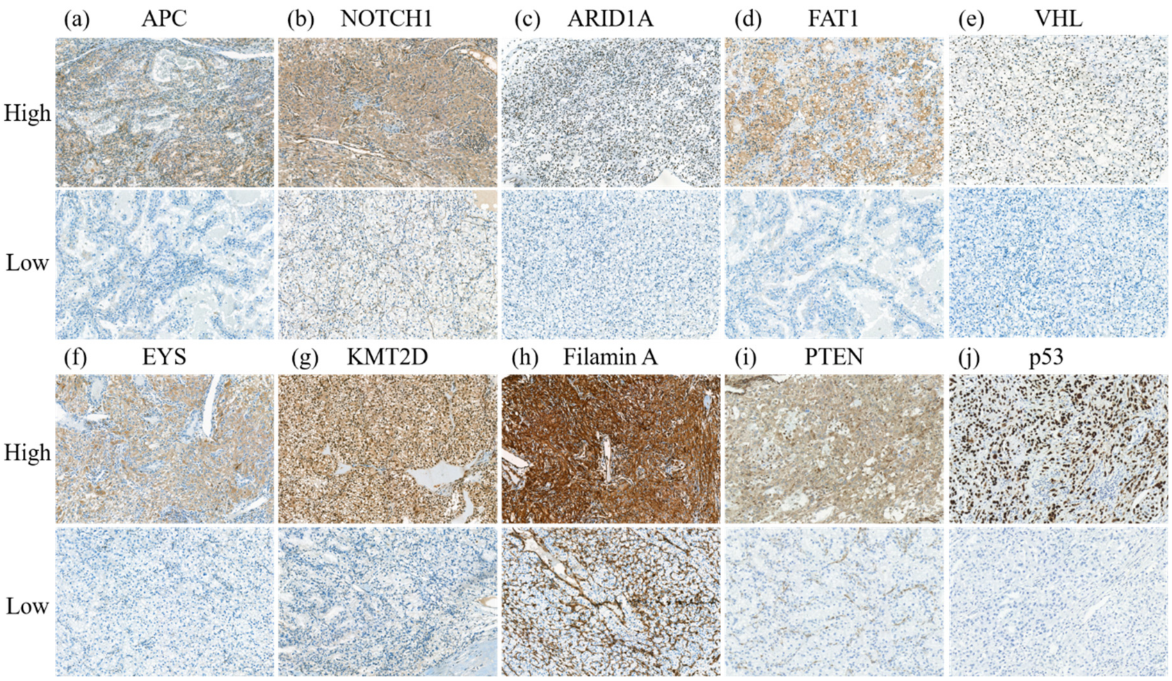

Figure S1. Representative images depicting high and low expression of APC (a), NOTCH1 (b), ARID1A (c), FAT1 (d), VHL (e), EYS (f), KMT2D (g), Filamin A (h), PTEN (i), and p53 (j) in clear cell renal cell carcinoma (x200). APC, NOTCH1, FAT1, EYS, Filamin A, and PTEN were expressed in the cytoplasm, and ARID1A, VHL, KMT2D, and p53 were expressed in the nucleus.

Publisher's Note: MDPI stays neutral with regard to jurisdictional claims in published maps and institutional affiliations.

© 2020 by the authors. Submitted for possible open access publication under the terms and conditions of the Creative Commons Attribution (CC BY) license (http://creativecommons.org/licenses/by/4.0/).
